# Defining Puberty and Spectrum of Hypogonadism in Alström Syndrome

**DOI:** 10.1210/clinem/dgaf356

**Published:** 2025-06-13

**Authors:** Sadaf Ali, Qianwen Zhang, Ijeoma Anwunah, Shanat Baig, Gabriela da Silva Xavier, Charlotte Dawson, Francesca Dassie, Yijun Tang, Libo Wang, Guoying Chang, Paul Gleeson, Adrian T Warfield, Richard Paisey, Timothy G Barrett, Pietro Maffei, Xiumin Wang, Victoria Homer, Melanie Kershaw, Tarekegn Geberhiwot

**Affiliations:** Department of Inherited Metabolic Disorders, Queen Elizabeth Hospital Birmingham, Birmingham B15 2GW, UK; Institute of Metabolism and Systems Research, University of Birmingham, Birmingham B15 2TT, UK; Department of Inherited Metabolic Disorders, Queen Elizabeth Hospital Birmingham, Birmingham B15 2GW, UK; Department of Endocrinology and Metabolism, Shanghai Children's Medical Centre, School of Medicine, Shanghai Jiao Tong University, Shanghai 200127, China; Birmingham Women's and Children's Hospital, Birmingham B4 6NH, UK; Department of Inherited Metabolic Disorders, Queen Elizabeth Hospital Birmingham, Birmingham B15 2GW, UK; Institute of Metabolism and Systems Research, University of Birmingham, Birmingham B15 2TT, UK; Institute of Metabolism and Systems Research, University of Birmingham, Birmingham B15 2TT, UK; National Institute for Health and Care Research (NIHR) Birmingham Biomedical Research Centre, Birmingham B15 2TH, UK; Department of Inherited Metabolic Disorders, Queen Elizabeth Hospital Birmingham, Birmingham B15 2GW, UK; Department of Medicine (DIMED), University of Padova, European Reference Network on Rare Endocrine Conditions (Endo-ERN), Azienda Ospedale-Università Padova, 35122 Padova, Italy; Department of Endocrinology and Metabolism, Shanghai Children's Medical Centre, School of Medicine, Shanghai Jiao Tong University, Shanghai 200127, China; Department of Endocrinology and Metabolism, Shanghai Children's Medical Centre, School of Medicine, Shanghai Jiao Tong University, Shanghai 200127, China; Department of Endocrinology and Metabolism, Shanghai Children's Medical Centre, School of Medicine, Shanghai Jiao Tong University, Shanghai 200127, China; Department of Imaging, Queen Elizabeth Hospital, Birmingham B15 2GW, UK; Department of Cellular Pathology, Queen Elizabeth Hospital, Birmingham B15 2GW, UK; Department of Diabetes and Endocrinology, Torbay and South Devon NHS Foundation Trust, Torbay TQ2 7AA, UK; Birmingham Women's and Children's Hospital, Birmingham B4 6NH, UK; Department of Cancer and Genomic Sciences, University of Birmingham, Birmingham B15 2TT, UK; Department of Medicine (DIMED), University of Padova, European Reference Network on Rare Endocrine Conditions (Endo-ERN), Azienda Ospedale-Università Padova, 35122 Padova, Italy; Department of Endocrinology and Metabolism, Shanghai Children's Medical Centre, School of Medicine, Shanghai Jiao Tong University, Shanghai 200127, China; Cancer Research UK Clinical Trials Unit, School of Medical Sciences, University of Birmingham, Birmingham B15 2TT, UK; Birmingham Women's and Children's Hospital, Birmingham B4 6NH, UK; Department of Inherited Metabolic Disorders, Queen Elizabeth Hospital Birmingham, Birmingham B15 2GW, UK; Institute of Metabolism and Systems Research, University of Birmingham, Birmingham B15 2TT, UK

**Keywords:** puberty, hypogonadism, Alström syndrome, reproduction, male infertility, primary testicular failure

## Abstract

**Context:**

Alström syndrome (AS) has been extensively studied for its multisystem organ manifestations. Primary gonadal failure is well described in humans, but little is known about the intricacies of puberty and true incidence of hypogonadism within this population.

**Objective:**

We aimed to define the onset and progression of puberty and the incidence of hypogonadism in male patients with AS.

**Methods:**

A retrospective, observational cohort study was conducted on patients with AS across the UK and Italy national services. Additionally, the findings were correlated with Alms1 S701X mouse model as part of the current study.

**Results:**

We enrolled 28 pediatric patients (age 14.8 ± 2.3) and 41 adult patients (age 34 ± 12). All pediatric patients entered puberty at an appropriate age, but the highest testicular volume achieved by patients with AS was 9 ± 3 mL in age group of 14- to 15-year-old boys. Among adults, 95% (39/41) had hypogonadism with primary gonadal failure. Testicular analysis of the Alms1 S701X mouse model shows testicular atrophy with no evidence of fibrosis. Moreover, Alms1 S701X mice exhibit reduced sperm count and sperm motility compared with controls (29.03 × 106/mL vs 110.6 × 106/mL, 34.77% vs 70.18%).

**Conclusion:**

Our study sheds light on the reproductive aspects of AS across pediatric and adult populations with particular emphasis on testicular and pubertal development, and hypogonadism in adult life. Although, all the pediatric patients with AS have age-appropriate onset of puberty, almost all exhibit hypogonadism with primary gonadal failure as adults. This mirrors the Alms1 S701X mouse model.

Alström syndrome (AS) is a rare autosomal recessive multisystem disease with an estimated prevalence of 1 per million of the population worldwide (1). It is caused by mutations in the *ALMS1* gene with various gene defects such as insertions, deletions, and nonsense mutations found primarily in exons 8, 10, and 16 (2). *ALMS1* encodes a 461-kDa protein of unknown function that has ubiquitous expression, hence affecting various organ systems (2, 3). Previous RNA knockdown experiments have shown impaired cilia formation, therefore linking it to ciliopathy (4). Studies have shown a subcellular localization of *ALMS1* in the centrosome and basal body as a likely mechanism to the pathogenesis of various organ manifestations seen in AS (5). It is a multisystem disease characterized by visual loss, childhood obesity, insulin resistance, type 2 diabetes, accelerated metabolic dysfunction–associated steatotic liver disease, hypertriglyceridemia, short stature, dilated cardiomyopathy, progressive renal dysfunction, and hearing loss (1, 2). In addition, endocrinopathies such as hypothyroidism, growth hormone deficiency and hypogonadism are well-known disease associations (6), with isolated case reports of empty sella syndrome, central adrenal insufficiency, and central diabetes insipidus (1, 2, 7-9).

Hypogonadism in AS is a recognized feature, but real-world data on patient cohorts are lacking. Evidence mainly comes from isolated case reports and cross-sectional data (1, 6). Puberty is thought to be often delayed with normal masculinization and development of secondary sexual characteristics in adult males (8). To date, there has been no systematic or longitudinal studies to define pubertal development and spectrum of hypogonadism in AS. Regular annual reviews of national cohorts of patients with AS in the UK and Italy facilitated this study to capture puberty and hypogonadism data in male subjects with AS. The study aimed to delineate the features of puberty and the spectrum of hypogonadism in AS cohorts, gathering data from detailed clinical assessments of pediatric and adult patients with AS over the past decade in the UK and Italy. The analysis sought to determine the incidence and etiology, and whether there has been any progression of hypogonadism over time in these cohorts of patients. Additionally, gonadal assessment and sperm characteristics were further explored in the *Alms1 S701X* mouse model to enhance the characterization of hypogonadism in AS.

## Materials and Methods

### Study Population for Human Studies

A retrospective observational exploratory study was conducted on patients with AS, utilizing data from 2 large European national reference centers for managing AS: University Hospitals Birmingham, Birmingham UK, and the Department of Medicine (DIMED) at the University of Padova, Padova Italy. All the UK and Italian patients with AS were seen in these 2 centers. The participants included in the study were all male patients over the age of 11 years with confirmed diagnosis of AS based on at least 2 pathogenic gene variants and major clinical diagnostic criteria. We excluded patients who did not have pathogenic gene mutations or only 1 pathogenic gene mutation, insufficient clinical information to infer information on puberty and hypogonadism, and all female patients with AS. Height SDS and height velocity data were analyzed in the Birmingham pediatric cohort. Patients were reviewed at each annual review clinic, and comprehensive assessments were carried out including detailed clinical assessment, blood tests, and imaging. The human study data were collected as part of a clinical service evaluation, utilizing secondary analysis of anonymized patient information. Therefore, study specific ethical approval was not required. The human postmortem (PM) studies were carried out with national research ethics committee approval (REC reference 10/H0203/33).

The study was performed according to international standards in agreement with the Declaration of Helsinki.

### Assessments

#### Clinical data

Electronic medical records were reviewed, and data collected for all pediatric and adult patients with AS who attended highly specialized national AS services based in the UK and Italy between 2006 and 2023. For pediatric patients, we obtained data on puberty by assessing pubic hair development, genital development, and testicular volume measured by Prader orchidometer using Tanner staging, and biochemical assessment of the gonadotrophic axis using total testosterone (TT), follicle-stimulating hormone (FSH), and luteinizing hormone (LH) levels. For the UK cohort of pediatric patients, height and body mass index (BMI) standard deviation scores (SDS) were obtained using The Royal College of Paediatrics and Child Health (RCPCH) growth charts. For the Italian cohort of pediatric patients, the weight and BMI SDS were established using the SIEDP (Italian Society of Pediatric Endocrinology and Diabetology) growth calculator (10).

Delayed puberty was defined as per the Tanner staging corresponding to the age, taking into account the testicular volume measurements, genitalia stage, pubic hair stage, and biochemical assessments (TT, FSH, LH) (11). Boys who achieved testicular volume of at least 4 mL (as measured by orchidometer) together with pubic hair stage 2 by the age of 14 were classified as entering puberty at an expected age (12, 13). Delayed puberty was considered in boys where testes remained less than 4 mL in volume by the age of 14 with pubic hair stage 1, genitalia stage 1, and TT less than 7 nmol/L (14). Arrested puberty was considered when there was lack of pubertal progression for more than 2 years after spontaneous onset and progression of puberty at an appropriate age (15). For adult patients with AS, weight was measured using a digital weighing machine in kilogram (kg) and height in meters (m) using a standing stadiometer. BMI was obtained by dividing body mass by square of the body height and expressed in units of kg/m^2^.

#### Biochemical assays

All patients underwent biochemical evaluation as part of their routine clinical care. Hormonal profile including LH, FSH, and TT were measured for all patients.

Pediatric measurements of basal LH and FSH were conducted using an immunoassay (Siemens DPC Immulite 2000 (16) and Roche Cobas e601 for the UK pediatric cohort and chemiluminescence for the Italian pediatric cohort). Testosterone measurement was carried out using liquid chromatography-tandem mass spectrometry for the UK cohort of pediatric and adult patients and immunoassay (chemiluminescence) for the Italian pediatric cohort. Samples were taken between 09:00 and 16:00. The reference range cutoff used according to Tanner stage were Tanner stage 1 testosterone <1 nmol/L, LH 0.7 to 2.6 U/L, FSH 0.2 to 4.0 U/L. Cutoff for raised gonadotrophins at Tanner stage 3 to 5 were LH >4 U/L, FSH >10.6 U/L (Table S1, data repository reference number 1288 (17)).

Normal hormone reference ranges for adult patients were LH 0.6 to 12.1 IU/L, FSH 1.0 to 12.0 IU/L, and TT 10 to 27 nmol/L. All adult patients had their fasting morning TT checked twice between 07:00 and 11:00, at least 6 months apart, to confirm testosterone deficiency.

For adult patients, as per the Society of Endocrinology guidelines (18), primary gonadal failure was defined as low fasting morning testosterone (TT <8 nmol/L, or TT at lower quartile of normal range) and high LH/FSH ratio. Secondary gonadal failure was defined as low testosterone (TT <8 nmol/L, or TT in the lower quartile of normal range) and normal, low, or undetectable LH.

#### Imaging

A testicular ultrasound (US) was performed in adult patients by an accredited ultrasonographer using Hitachi Ascendus, 14-6 MHz for structural assessments of gonads. The testicular volume was calculated in adult patients with AS utilizing US measurements by applying the ellipsoid formula (length × width × thickness × 0.52), which has been widely used before (19).

### In Vivo Mouse Studies

#### Mouse model

Animal studies were performed in accordance with the recommendations in Australian Code of Practice for the care and use of animals for scientific purposes of the National Health and Medical Research Council. Animal studies were performed in China and all experimental protocols were approved by the Institutional Animal Care and Use Committee at Shanghai Jiao Tong University, School of Medicine (JUMC2023-096-A). Mice were housed at a controlled temperature (25 °C), in pathogen-free conditions, and a 12-hour light/dark cycle with free access to rodent food and water. The Alms1 S701X mouse model was generated using a CRISPR-Cas9 approach, leading to the termination of protein translation in serine 701 (Gempharmatech Co., Ltd), which represented the hot spot mutation p.S697X in Chinese patients and displayed similar symptoms to patients with AS (Fig. S1 and 2, data repository reference number 1288 (17)). Primers flanking the mutation were used for PCR and Sanger sequencing: forward, TTACCACCAAGAGTTGCCAGACAG; reverse, CTGGTCTACTGTTCCAGTTGTACCC.

### Histology

For histology, the testes were infiltrated with 4% paraformaldehyde solution and fixed overnight at room temperature. After embedding in paraffin, testes were sectioned at a thickness of 4 μm for hematoxylin and eosin (H&E) and Masson's trichrome staining using standard protocols.

### Measurement of Testosterone

Peripheral blood was collected from the orbital venous plexus of anesthetized 8-week-old mice using isoflurane. Blood samples were allowed to clot at room temperature for 30 minutes before centrifugation (15 minutes, 12 000 rpm, 4 °C). The serum was then stored at −80 °C until analysis. Testosterone was measured in 100 μL of serum on a Thermo Q Exactive plus mass spectrometry (20). Experiments were performed using Aligent ZORBAX SB-aq column (2.1 × 50 mm, 1.8 μm) and mobile phase solvents consisting of (1) 0.06% acetic acid in water and (2) acetonitrile with 0.06% acetic acid. Metabolites were quantified by calculating the peak area.

#### Collection of sperm

Epididymal cauda sperm were collected via swim-out (21, 22). Testicular sperm analyzed in this study were collected from 8-week-old male *Alms1 S701X/S701X* (Mut) mice or male littermate control (wild type [WT]). Briefly, cauda epididymides were dissected and placed in 1 mL of EmbryoMax Human Tubal Fluid (MR-070-D). Sperm were allowed to swim-out for 20 minutes at 37 °C and then the epididymides were removed.

#### Sperm analyses

Sperm suspensions (10 µL) were introduced into a prewarm Leja slide (100 µm thick) and maintained at 37 °C during recording. Sperm motility was examined using the CEROS computer-assisted semen analysis system (SAS-AM-II) (23). At least 5 microscopy fields were analyzed in each experiment.

### Statistical Analysis

Descriptive statistics were used to summarize baseline characteristics. Summary statistics are presented as mean and SD or standard error of the mean (SEM). Comparison was performed by t-test or nonparametric testing based on the data types and distributions. Hierarchical linear models were used to quantify the relationship between age at clinical assessment and standardized height in pediatric patients. Group level terms were added to account for the lack of independence in measurements. Statistical analyses were performed using Excel, SPSS statistical software, version 26, GraphPad Prism 5 software (GraphPad Software, Inc.), and R version 4.2.0.

## Results

### Baseline Characteristics of Study Participants

**Figure 1.**
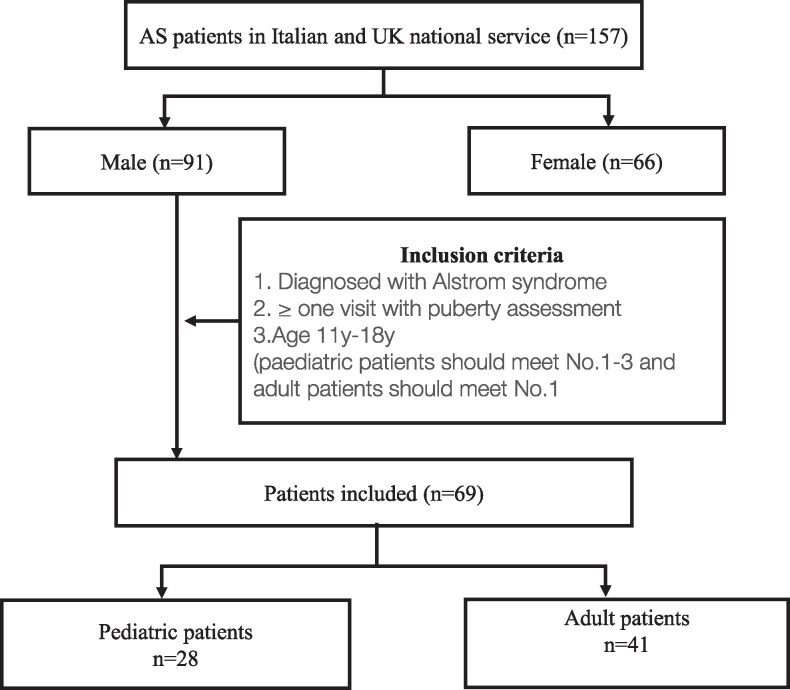
Study cohort flow diagram.

As shown in Fig. 1, 157 patients (91 males, 66 females) from the 2 national services were screened for this observational study. Of these, 69 male patients, who were seen in their respective AS follow up clinics in the UK and Italy between 2006 and 2023, fulfilled inclusion and exclusion criteria for this study (Fig. 1). All 69 participants (28 pediatric patients [age 11-18] and 41 adult patients) had disease causing pathogenic variants in the *ALMS1* gene and met clinical diagnostic criteria for AS. Anthropometric, clinical, and metabolic characteristics are shown in Table 1. The mean age of pediatric cohorts was 14.8 (±2.3) years, while adults had a mean age of 34 (±12). The prevalence of type 2 diabetes was 35% and 76% in the respective pediatric and adult cohorts.

**Table 1.** Baseline characteristic of pediatric and adult patients with Alström syndrome

Characteristics (mean ± SD)	Group	
	Pediatric patients (n = 28)	Adult patients (n = 41)
Age (years)	14.8 ± 2.3	34 ± 12
Height(m)*^a^*	1.58 ± 10.47*^b^* (−1.00 ± 1.63)	1.62 ± 0.07
Weight(kg)*^a^*	65 ± 18*^b^* (0.81 ± 1.20)	78 ± 18
BMI (kg/m^2^)*^a^*	27 ± 5*^b^* (1.41 ± 0.89)	31 ± 6
Diabetes, % (n)	35% (10)	76% (31)
HTN, % (n)	32% (9)	61% (25)
CKD, % (n)	—	39% (11)
Cardiomyopathy % (n)	—	43% (12)

Abbreviations: BMI, body mass index; CKD, chronic kidney disease; HTN, hypertension.

^*a*^Recorded at last clinic visit.

^*b*^Standard deviation scores.

### Puberty Assessment in Pediatric Patients

**Figure 2.**
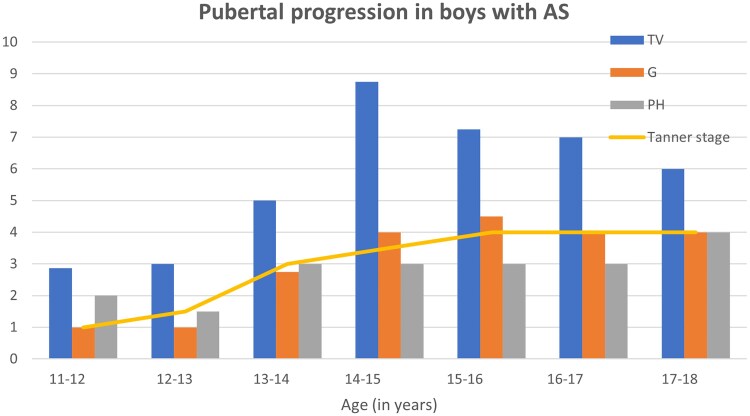
Bar charts showing pubertal progression in boys with AS with an age range of 11-18. TV, testicular volume in milliliters; G, genital Tanner stage (showed as Tanner stage 1-4); PH, pubic hair tanner stage (showed as Tanner stage 1-4).

Longitudinal pubertal development in boys aged 11-18 (Table 2 and Fig. 2) was assessed through markers such as testicular volumes, genital development, pubic hair development, and Tanner staging. The data demonstrated a progressive increase in Tanner stage from age 11 to 16, with most boys progressing from Tanner stage 1 at ages 11-13 through to stages 3 and 4 by age 14-16. Notably, after age 16, puberty appeared to stop progressing beyond Tanner stage 4, with boys remaining at this stage through ages 17 and 18. Genital and pubic hair development followed a similar trend, with significant changes occurring around ages 13-15, and then staying at Tanner stage 4 after 16 years of age

**Table 2.** Pubertal development in Alström syndrome

Clinical characteristics	11-12 years (n = 13)	12-13 years (n = 16)	13-14 years (n = 16)	14-15 years (n = 13)	15-16 years (n = 13)	16-17 years (n = 10)	17-18 years (n = 6)
Weight SDS	1.46 ± 0.74 (92%)	1.07 ± 0.92 (81%)	0.84 ± 1.40 (94%)	1.30 ± 0.69 (77%)	1.30 ± 0.73 (100%)	0.82 ± 0.92 (90%)	0.15 ± 0.78 (83%)
BMI SDS	1.56 ± 0.74 (92%)	1.51 ± 0.68 (81%)	1.29 ± 1.07 (94%)	1.84 ± 0.53 (77%)	1.79 ± 0.41 (100%)	1.66 ± 0.46 (90%)	1.80 ± 0.51 (83%)
Physical examination							
RTV (mL)^*a*,*b*^	3, 4.1-2 (92%)	3, 5.12-2.25 (75%)	5, 8-4.5 (75%)	9.5, 10-6 (54%)	8, 13-4 (46%)	7, 8-7 (60%)	6, 7.5-4.5 (67%)
LTV (mL)^*a*,*b*^	2.75, 3.75-2 (92%)	3, 4.5-2 (75%)	5, 6.5-4.5 (75%)	8, 9-5 (46%)	6.5, 11.75-3.5 (38%)	7, 8-7 (60%)	6, 7.5-4.5 (67%)
Genital development (Tanner stage)*^b^*	1, 1.25-1 (85%)	1, 1.25-1 (75%)	2.75, 3-2.13 (81%)	4, 4.25-3.75 (62%)	4.5, 5-3.75 (46%)	4, 4-4 (44%)	4,4-4 (83%)
Pubic hair development (Tanner stage)*^b^*	2, 2-1.5 (77%)	1.5, 2-1 (75%)	3, 3-2.75 (69%)	3, 3-3 (54%)	3, 3-3 (38%)	3, 4-3 (60%)	4,4-4 (83%)
Tanner stage	1	1-2	3	3-4	4	4	4
Laboratory result							
TT (nmol/L)	1.1 ± 1.1 (54%)	4.02 ± 3.33 (56%)	7.18 ± 7.22 (63%)	7.08 ± 2.15 (69%)	8.3 ± 4.68 (46%)	7.94 ± 3.73 (60%)	8.87 ± 2.59 (67%)
LH (IU/L)	1.27 ± 1.04 (46%)	1.92 ± 1.77 (56%)	4.49 ± 3.70 (63%)	9.91 ± 5.95 (62%)	14.95 ± 7.67 (46%)	15.75 ± 9.88 (60%)	20.23 ± 6.52 (67%)
FSH (IU/L)	2.33 ± 2.03 (46%)	4.87 ± 3.44 (56%)	6.30 ± 4.84 (63%)	13.89 ± 8.707 (62%)	18.53 ± 17.31 (46%)	19.15 ± 17.06 (60%)	26.88 ± 23.27 (67%)

Values presented as mean ± SD (percentage of patients with available data).

Abbreviations: BMI, body mass index; FSH, follicle stimulating hormone; FT, free testosterone; LH, luteinizing hormone; LTV, left testicular volume; NA, not available; RTV, right testicular volume; SHBG, sex hormone–binding globulin; TT, total testosterone.

^*a*^Testicular volume measured by orchidometer.

^*b*^Median, IQR (percentage of patients with available data).

Normal reference ranges of testicular volume measured by Prader orchidometer for adults achieving puberty at normal rate, adapted from Goed et al (15); age 11-12: R testis 4.67 ± 2.89, L testis 4.5 ± 2.75; age 13-14: R testis 12.89 ± 6.74, L testis 12.4 ± 6.35; age 15-16: R testis 22.01 ± 4.41, L testis 21.64 ± 4.35; age 17-18: R testis 24.92 ± 5.45, L testis 25.1 ± 3.94. Reference range cutoff used according to Tanner stage 1 testosterone < 0.7 nmol/L, LH 0.7-2.6 U/L, FSH 0.2-4.0 U/L. Cutoff for raised gonadotrophins at Tanner stage 3-5 LH > 4 U/L, FSH > 10.6 U/L (liquid chromatography-mass spectrometry method).

**Figure 3.**
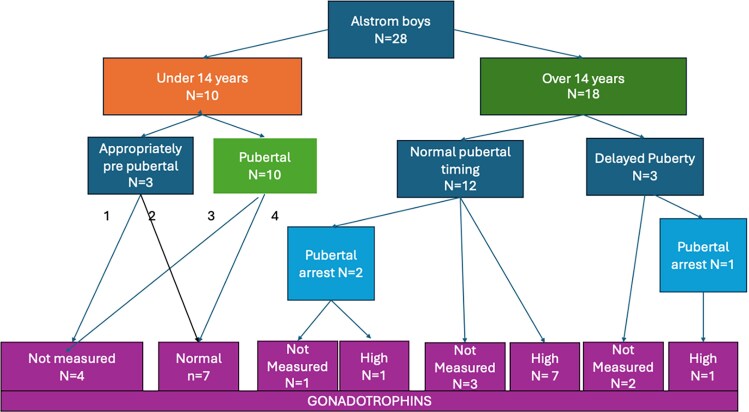
Pubertal onset and progression in boys with AS taking into account clinical data and gonadotrophin levels (Birmingham, UK pediatric cohort).

Biochemical markers of LH, FSH, and testosterone, as a proxy, together with the clinical data were used to determine likely onset of puberty in the Birmingham UK pediatric cohort. Three of 10 boys under 14 years were normally prepubertal for age, and 7 of 10 boys under 14 years at last assessment were already pubertal (median age puberty confirmed 12.8 years, range 11.6-13.9 years; Fig. 3).

Of 18 boys over 14 years at their last assessment, 3 had delayed puberty (*X*^2^ (1, n = 21) = 3.6, *P* = .57 NS), 2 boys having entered puberty at 14.5-15.5 years with subsequent arrested pubertal development, and 1 remained prepubertal age 16.5 years. In 3 older pubertal patients over 14, the timing could not be ascertained, but puberty was confirmed at 16.5 years clinically and biochemically, and at 15.1 years and 18.9 years biochemically in the remainder. Of the remaining boys with normal onset of puberty, 2 had arrested puberty, 1 had high gonadotrophins, and the other had no gonadotrophin results available. Of those with apparently normal pubertal progression, gonadotrophins were raised in 7/12.

Levels of LH and FSH remained high at the age of 18 with mean LH of 20.23 ± 6.52 IU/L and FSH of 26.88 ± 23.27 IU/L (Table 2) compared with an average level of LH 3.6 ± 0.6 and FSH 20.2 ± 6.1 in a population based study conducted in healthy male children and adolescents at age of 18-20 (24).

**Figure 4.**
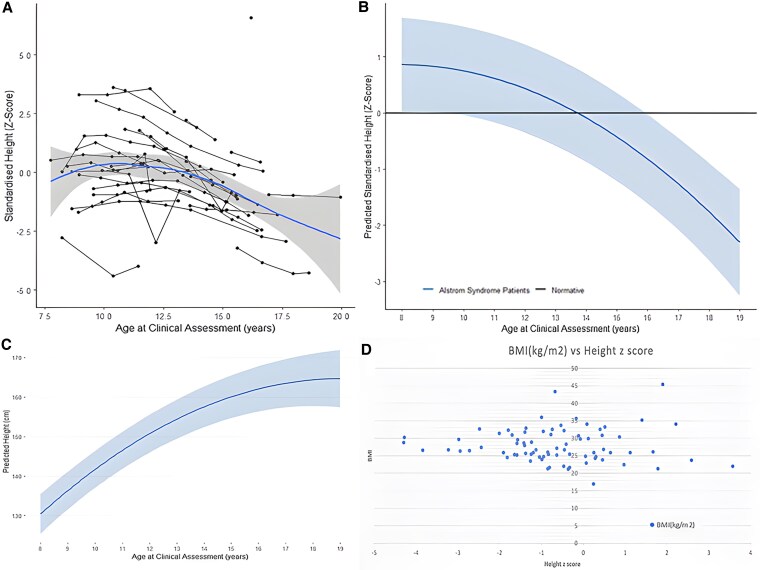
Height z score by age and BMI in boys with AS. (A) Standardized height z score by age, with black lines indicating patient group studied and dark blue thick line with shaded band behind it depicting the best fit. (B) Predicted standardized height z score to age (years); (C) Predicted height (cm) to age (years). (D) Correlation of height z score with BMI.

**Figure 5.**
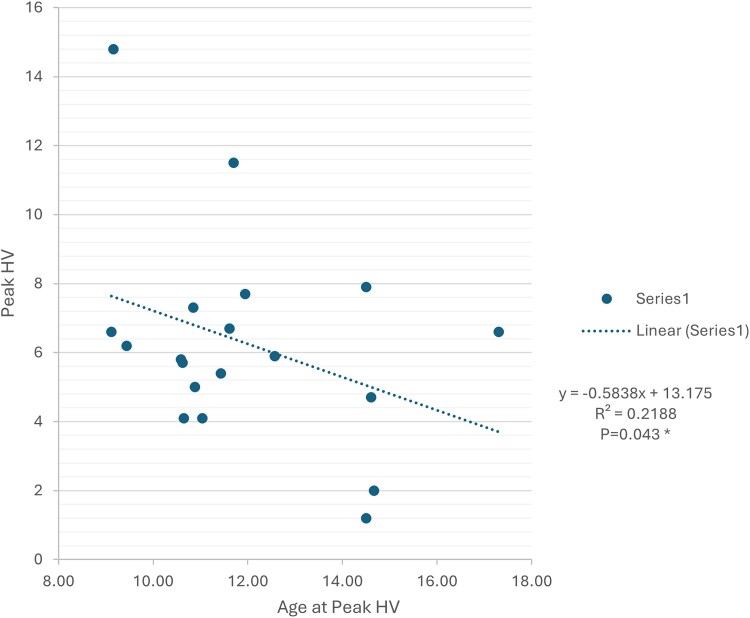
Correlation between age at peak height velocity and peak height velocity achieved in boys with AS with normal onset of puberty.

We analyzed height z score by age and BMI and found negative correlation with height z score decreasing to less than 0 after age 13, and no correlation between BMI and height z score in boys of pubertal age (Fig. 4). Mean height z score was >1 in prepuberty and early puberty; however, 1 in 5 boys had a last recorded height z score of less than −2. There was no correlation between peak height velocity (PHV) or age at PHV with the last recorded height in those >14 years with normal pubertal timing (Fig. 5). PHV achieved in boys with AS was significantly negatively correlated with age at PHV, as seen in healthy boys (*P* = .0024). This remained significant when boys with delayed puberty were excluded from the analysis (*P* = .043). Mean age at recorded PHV was 11.8 years (range 9.1-17.3 years) and significantly different to the healthy population where mean age at PHV is 14.0 years (*P* < .0001, CI 1.7-2.7 t test). Mean achieved PHV was 5.5 cm/year and significantly lower than the mean PHV of 9.5 cm/year in healthy boys (*P* < .0001, CI 3.67-4.62, t test).

At age 9, there was no significant difference between the height of the patients with AS and that of the standardized age-matched population (*P* = .098), although the mean height z score was >0. As age increased, there was a significant decrease in height compared with the standardized age-matched score (*P* < .001). Height z score then progressed as shown in Fig. 4B (mean, 95% CI). Figure 4C shows an estimate of how height would usually progress for an AS patient during puberty in the population. There was no difference in the pattern of height velocity centiles recorded pre 12 years between those whose last recorded height z score was less than −2 SDS (severe short stature) and those with height z score greater than −2 SDS. In addition, height velocities for age were frequently recorded as above the 50th centile for age, even in those who subsequently had severe short stature.

### Adult Hypogonadism

As shown in Table 3, 39 (95%) adult patients had hypogonadism with primary gonadal failure, yet less than a quarter of adult patients (22%) were on testosterone replacement therapy. Combining data of gonadal assessment using US (14/41 adult patients or 34% of cohort) and postmortem (PM) examination (4/41 adult patients or 7% of cohort), we infer that all adult patients with hypogonadism either had testicular atrophy (86%) or absent testes (14%). Estimation of testicular volume based on US using the ellipsoid formula showed an average volume of 1.71 ± 0.90 mL in right testis and 1.88 ± 1.24 mL in left testis in adult patients with AS (Table S2, data repository reference number 1288 (17)). Of note, the PM of a 40-year-old patient (Table 3, patient 3) showed prepubertal external genitalia (Tanner male stage 1) and a micropenis which was markedly hypoplastic.

**Table 3.** Characteristics of hypogonadism in adult patients with Alström syndrome

Characteristics (n = 41)	Values mean ± SD
Hypogonadism, % (n)	95 (39)
**Spectrum of hypogonadism**	
Primary gonadal failure, % (n)	100 (39)
On replacement, % (n)	22 (9)
TT < 8 nmol/L,*^a^* % (n)	60 (18)
TT 8-12 nmol/L,*^a^* % (n)	30 (9)
TT 13-14 nmol/L,*^a^* % (n)	10 (3)
**Laboratory result**	
TT (nmol/L)*^a^*	7.41 ± 3.96
LH (IU/L)*^a^*	16.72 ± 9.63
FSH (IU/L)*^a^*	25.6 ± 15.46
**Testes measurement**	
Ultrasound (n = 14)	
Testicular volume (mL)*^c^*	Right 1.7 ± 0.90
	Left 1.8 ± 1.24
**Postmortem**	
Patient 1*^b^*	
Age at death	58
Cause of death	Massive pulmonary embolism
LH	<0.5
FSH	<0.5
TT	16.6
Testes	The scrotum contained a single hypoplastic testis (18 g). No viable second cryptorchid testis was identified
Patient 2*^b^*	
Age at death	39
Cause of death	Community-acquired pneumonia/sepsis
LH	<0.5
FSH	<0.5
TT	12.9
Testes	Both testes visualized (left 22 g, expected weight 25.4 g; right 19 g, expected weight 24.7 g)
Patient 3*^b^*	
Age at death	40
Cause of death	Community-acquired pneumonia/sepsis
LH	<0.5
FSH	<0.5
TT	49.2
Testes	Genitalia were prepubertal (Tanner male stage 1). The noncircumcised micropenis was markedly hypoplastic. The rudimentary scrotum was empty—neither testis was identifiable after targeted prosection of sites of incomplete/mal descent, including retroperitoneum, pelvic cavity, inguinal canal, and scrotum.

Abbreviations: BMI, body mass index; FSH, follicle-stimulating hormone; LH, luteinizing hormone; SHBG, sex hormone–binding globulin; TT, total testosterone

^*a*^n = 30/39, not on testosterone replacement therapy.

^*b*^On testosterone therapy. TT reference range 10-27 nmol/L; LH reference range 0.6-12.1 IU/L; FSH reference range 1.0-12.0 IU/L; SHGB reference range 13.5-71.4 nmol/L.

^*c*^Testicular volume is presented as mean and SD (n = 10 for right testes and n = 9 left testes).

### Characteristics of Testes of Alms1S701X Mice

**Figure 6.**
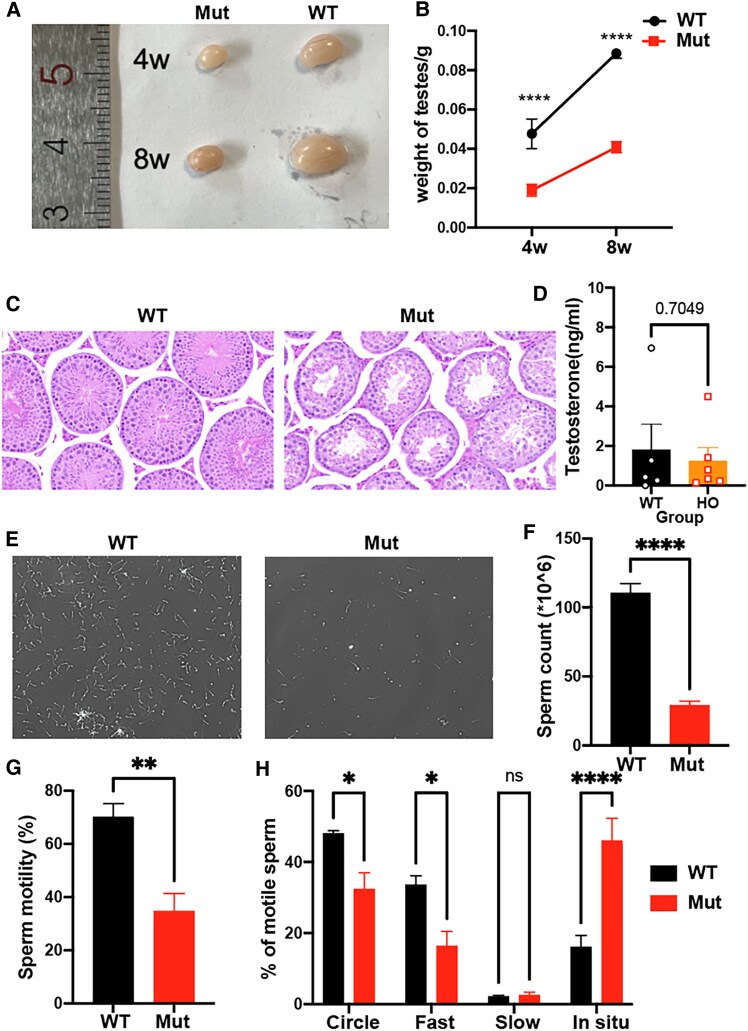
Testicular and sperm analysis in the male mouse model of Alström syndrome. (A) Representative images of testis from 4-week-old (4W) and 8-week-old (8W) WT and Mut mice respectively. (B) Weight of testes from 4-week-old and 8-week-old WT and Mut mice respectively (n = 5 WT; n = 6 Mut). (C) Representative micrographs of H&E-stained testicular sections of 8-week-old mice. Scale Bar = 20 µm. (D) Level of testosterone in serum from 8-week-old WT and Mut mice respectively (n = 6 WT; n = 6 Mut). (E) Representative images of living sperm cells from 8-week-old mice by the computer-assisted semen analysis (CASA) system device. (F-H) Sperm count per mL, percentage of total motile sperm in the population, and classification of type of motility out of the total motile population was examined using the CASA system (n = 4 WT; n = 6 Mut). Values represent the mean ± SEM. *P < .05, **P < .01, ****P < .0001.

To widen the understanding of hypogonadism in AS, we carried out testicular analysis in *Alms1 S701X/S701X* mutant mouse model (Mut) and compared it with littermate WT mice. The testicular size and weight in Mut mice were significantly lower than WT, both at 4 weeks and 8 weeks of age (Fig. 6A and 6B). Histological analysis of testes using H&E stain in Mut mice in comparison to WT showed disorganized seminiferous tubules with fewer germs cells indicating a lack of effective germ cell development and failure of spermatogenesis (Fig. 6C). There was no significant difference in serum testosterone between Mut and WT mice (Fig. 6D). Eight-week-old male Mut mice had lower sperm counts (29.03 × 106/mL vs 110.6 × 106/mL) with reduced motility (34.77% vs 70.18%) vs WT mice. The percentage of circle and fast motile sperms was lower in Mut mice vs the WT mice (Fig. 6E-6H).

**Figure 7.**
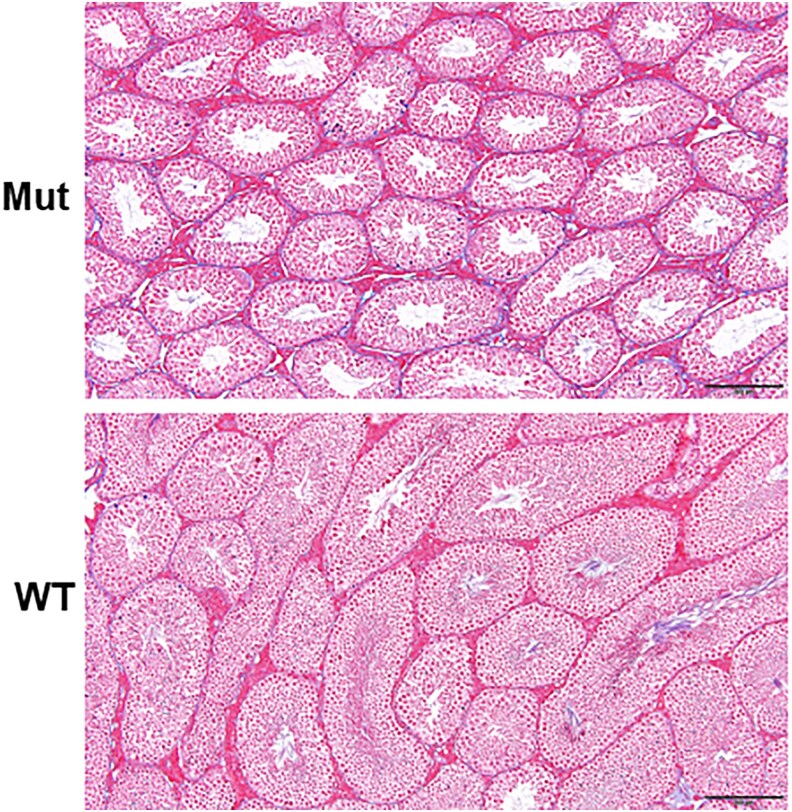
Masson staining of testis from 24-week-old *Alms1 S701X/S701X* (Mut) and control (WT) mice. Scale Bar = 200 µm, with no evidence of fibrosis in *Alms1 S701X/S701X* mice.

Masson's trichrome staining in 24-week-old mice (Fig. 7) showed that there was no evidence of fibrosis in the testis of Mut mice.

## Discussion

This is the first in-depth large AS cohort study to gain mechanistic insight into the onset of puberty and spectrum of hypogonadism in pediatric and adult male patients with AS. In our multinational cohort, boys with AS entered and progressed through puberty at an expected age, but none of the boys completed the pubertal maturation, and many had biochemical evidence of hypergonadotrophinemia. In adult males with AS, hypogonadism was nearly ubiquitous, with primary gonadal failure accounting for 95% of the studied adult cohort. In our study, all adult patients who underwent gonadal assessment, either through imaging or PM examination, displayed atrophied or absent/undescended testes. Additionally, the absence of progeny among our group of 41 adult male patients, in conjunction with findings in the literature, highlights a potential challenge related to infertility in males with AS.

We reviewed longitudinal data of pubertal assessment over several years in a large cohort of patients with AS. Our analysis indicates that most male patients with AS had an appropriate onset of puberty with an average age of 13.49 ± 1.49. However, the progression of puberty follows a typical pattern, with testicular growth and the development of secondary sexual characteristics advancing until about age 16, at which point many boys reach Tanner stage 4 and puberty ceases to progress further, remaining at this stage through age 18. In adults with median age of 29.5 (IQR 43-23.75) primary gonadal failure is a universal finding, likely indicating that once puberty reaches Tanner stage 4 there is pubertal arrest followed by primary testicular failure into adult life. These findings contrast with previous cross-sectional studies in this cohort, which reported a tendency for delayed puberty, with adult males typically exhibiting preserved masculinization and secondary sexual characteristics (6). Furthermore, we observed a declining trend in height z scores among patients with AS during puberty, suggesting a potential association with impaired pubertal development. Pubertal growth depends on upon secretion of growth hormone, thyroid hormone, and sex hormones (androgens and estrogens) (25). Sex hormone secretion leads to fusion of growth plates and subsequent cessation of growth (26). Hypogonadism in adolescence is usually associated with a delayed bone age and a prolonged growth period in adolescence associated with low androgen hormones. Bone age was not assessed in this cohort, but this warrants further evaluation in this patient group. We have demonstrated that boys with AS have a higher than expected predicted height z score between 8 and 12 years (Fig. 4 B). This could be driven by childhood obesity or relatively early puberty as seen with relatively early PHV. There is then an observed fall off predicted height z score during puberty. Age at PHV and mean PHV achieved was lower than the healthy population. Height z score can shift during puberty depending on pubertal timing. In earlier than average puberty, one expects to see a rise in height z score and then a fall, whereas in those with later puberty a fall-off in z score is followed by a rise. However, height z score at end of puberty in healthy males would normally be expected to be close to height z score prepubertally, but this was not the case in this cohort. This is a particularly difficult cohort to evaluate, and consideration should be given to recommending closer monitoring of height velocity and pubertal staging.

In a cross-sectional study of 35 adult males with AS, hypogonadism was reported in 77% despite normal masculinization and presence of secondary sexual characteristics. Both hypergonadotropic and hypogonadotropic hypogonadism have been reported in individuals with AS, with primary gonadal failure being a prevalent manifestation (6). In another observational study of 18 male patients with AS, Han et al (8) report hypogonadism in 57% of adult males with 36% having primary gonadal failure with elevated gonadotrophins. This was consistent with impaired genital maturation such as microphallus (50%), undescended testes (11%), hypospadias (5.6%), and small testes in adulthood (100%). This contrasts with our large longitudinal study wherein 95% (39/41) of all adults demonstrated primary gonadal failure. It is interesting to note that in 2 adult patients (2/41, 5%), did not show hypogonadism based on their testosterone levels. However, in one of these patients, testosterone was in the mid normal range, but with gonadotrophin levels were elevated-suggestive of impending gonadal failure.

Our comprehensive longitudinal study, conducted over several years, indicates that primary gonadal failure is an almost universal occurrence in adult males with AS. This was corroborated by evaluating testicular size either by US or PM analysis. All patients who underwent testicular US exhibited either testicular atrophy or absent/undescended testis. Similarly, the 3 patients who underwent PM assessment showed similar findings. This observation is consistent with previous published studies where adult patients with AS had either testicular atrophy or undescended testis (8). Comparable outcomes with our mouse study were noted in previous studies carried out on Alms1 foz/foz male mice, indicating testicular atrophy in comparison to wild-type mice (27).

The notable prevalence of absent or undescended testis (14%) in our study, underscores the necessity for further research, suggesting a potential crucial role of ALMS1 protein in gonadal development. Additionally, undescended testes not only have detrimental effects on male reproductive health but also has a 4- to 5-fold increased risk of testicular cancer (28). Hence, a comprehensive assessment is of paramount importance.

Based on the mouse models (*Alms1^Gt (XH152) Byg,^ Alms1^foz^* (fat aussie), *Alms1^L2131X^*), previous reports have suggested gonadal tissue fibrosis as a contributing factor to primary gonadal failure in adult patients with AS. These models have demonstrated atrophic seminiferous tubules, progressive germ cell loss, and various defects in sperm formation and motility (27, 29). It is important to note that AS has historically been recognized as a multiorgan fibrotic disorder. However, our mouse model (*Alms1 S701X*) testicular histology confirms the absence of fibrosis. This coincides with findings in recent years, where we and others have shown that fibrosis is neither intrinsic to AS nor universal to its target organ (1, 30-32). Instead, it appears to be a consequence of long-term metabolic and hypoxic insults to the organs.

The molecular mechanisms underlying the nearly universal finding of primary gonadal failure in AS remain unknown. *ALMS1* is ubiquitously expressed throughout all organ tissues, especially in the brain, lung, heart, kidney, large intestine, spleen, eye, ovary, and testis (33). Data from BioGPS shows a very high expression of the *ALMS 1* gene in the Leydig cells in humans, higher than all other human tissues (34). The expression of the *ALMS1* gene has been shown to be associated with testicular development in horses (35). Hence, it is plausible that *ALMS1* plays a role in regulating the development of Leydig cells, and mutations in this gene may have implications for testicular development. In our study, this is exemplified by smaller average testicular volume relative to age in individuals with AS compared with healthy individuals, as well as higher rate of testicular atrophy or absent testes in AS.

To date, there have been no reported cases of fertility in adult male patients with AS, in contrast to females (36). Detailed analysis of gonadal function and spermatogenesis in male fat aussie mice shows impaired spermatogenesis (27). The sperm analysis in 8-week-old male HOMO mice in our study revealed altered motility and reduced sperm counts. Furthermore, among all adult male patients with AS under our care, none have fathered children. This suggest that male hypogonadism and infertility may be a fundamental aspect of the expanding spectrum of AS. Nonetheless, a more in-depth investigation into the reproductive function of adult male patients with AS is essential, particularly if fertility is desired.

Limitations of the current study include, data collection limited to boys within the 11-18 age range, though PHV data collection for these boys was sought and analyzed from age 8 years in UK cohort of pediatric patients with AS. Additionally, we have not been able to analyze a seminiferous tubule biomarker, such as inhibin B or AMH, during pubertal maturation as data were extracted from routine clinical care records and these markers do not form part of routine clinical care. Additionally, in our study, we were unable to explore the clinical features associated with low testosterone levels or primary gonadal failure, nor did we have data on external genitalia assessment in adult males. However, moving forward, these assessments will be integrated into routine clinical care as part of subsequent, more comprehensive studies. The strength of the study is the longitudinal nature of the data gathered from the 2 largest AS service centers in the world.

To conclude, our study sheds light on the complex spectrum of puberty and hypogonadism in AS, revealing distinct patterns in both pediatric and adult cohorts. While a significant proportion of pediatric patients entered puberty normally, the compromised testicular volumes underscore the unique challenges associated with AS. Furthermore, the decline in height z score during puberty prompts important questions regarding potential compromises in pituitary growth hormone secretion and whether medical management of puberty could influence pubertal growth. Among adults, the overwhelming prevalence of hypogonadism, primarily characterized by primary gonadal failure, highlights the critical need for targeted interventions. The absence of fatherhood in adult participants and the findings in lower organisms further emphasize the reproductive implications of AS. These comprehensive insights into the reproductive aspects of AS contribute valuable knowledge for guiding clinical management and fostering a deeper understanding of this rare genetic disorder.

## Data Availability

Some or all datasets generated during and/or analyzed during the current study are not publicly available but are available from the corresponding author on reasonable request.

## Abbreviations

AS: Alström syndrome
BMI: body mass index
FSH: follicle-stimulating hormone
LH: luteinizing hormone
PHV: peak height velocity
PM: postmortem
TT: total testosterone
US: ultrasound
WT: wild type
